# Minimizing bias in massive multi-arm observational studies with BCAUS: balancing covariates automatically using supervision

**DOI:** 10.1186/s12874-021-01383-x

**Published:** 2021-09-20

**Authors:** Chinmay Belthangady, Will Stedden, Beau Norgeot

**Affiliations:** Anthem AI, Palo Alto, CA 94301 USA

**Keywords:** Causal inference, Observational studies, Neural networks, Deep learning

## Abstract

**Background:**

Observational studies are increasingly being used to provide supplementary evidence in addition to Randomized Control Trials (RCTs) because they provide a scale and diversity of participants and outcomes that would be infeasible in an RCT. Additionally, they more closely reflect the settings in which the studied interventions will be applied in the future. Well-established propensity-score-based methods exist to overcome the challenges of working with observational data to estimate causal effects. These methods also provide quality assurance diagnostics to evaluate the degree to which bias has been removed and the estimates can be trusted. In large medical datasets it is common to find the same underlying health condition being treated with a variety of distinct drugs or drug combinations. Conventional methods require a manual iterative workflow, making them scale poorly to studies with many intervention arms. In such situations, automated causal inference methods that are compatible with traditional propensity-score-based workflows are highly desirable.

**Methods:**

We introduce an automated causal inference method BCAUS, that features a deep-neural-network-based propensity model that is trained with a loss which penalizes both the incorrect prediction of the assigned treatment as well as the degree of imbalance between the inverse probability weighted covariates. The network is trained end-to-end by dynamically adjusting the loss term for each training batch such that the relative contributions from the two loss components are held fixed. Trained BCAUS models can be used in conjunction with traditional propensity-score-based methods to estimate causal treatment effects.

**Results:**

We tested BCAUS on the semi-synthetic Infant Health & Development Program dataset with a single intervention arm, and a real-world observational study of diabetes interventions with over 100,000 individuals spread across more than a hundred intervention arms. When compared against other recently proposed automated causal inference methods, BCAUS had competitive accuracy for estimating synthetic treatment effects and provided highly concordant estimates on the real-world dataset but was an order-of-magnitude faster.

**Conclusions:**

BCAUS is directly compatible with trusted protocols to estimate treatment effects and diagnose the quality of those estimates, while making the established approaches automatically scalable to an arbitrary number of simultaneous intervention arms without any need for manual iteration.

## Background

The ability to identify causal variables and estimate their impact is a critical task across many industrial and scientific fields [1–5]. Causal inferences can be drawn from two, generally distinct, types of analytical methods: randomized experiments and observational studies. Randomized Controlled Trials (RCTs), such as FDA trials to approve new drugs, are the gold standard for causal inference but they are not without shortcomings. Their rigor is often associated with high costs and therefore necessitates relatively small cohorts and short trial durations and may feature surrogate endpoints. Observational studies are increasingly being used to provide supplementary evidence in addition to RCTs [6, 7] because they provide a scale and diversity of participants and outcomes that would be infeasible in an RCT. Additionally, observational studies more closely reflect the settings in which the studied interventions will be applied in the future. In many application areas there is therefore increasing interest in using observational studies to complement randomized experiments to determine which actions are likely to produce the most desired outcome for a given circumstance or patient.

While observational studies have advantages, they present a distinct set of challenges. In particular, the effects of interventions may be confounded by variables that affect both treatment assignment as well as treatment outcome. Well-established techniques exist to control for cofounding variables in observational studies and estimate causal treatment effects. The propensity-score method pioneered by Rubin and Rosenbaum [8], and its many extensions such as inverse probability of treatment weighting (IPTW) [9] and Double-Robust (DR) [10] estimation, is widely used in medical research, healthcare, and epidemiology [11]. In addition to providing causal estimates, these methods also provide quality assurance diagnostics that assess balance in covariate distributions between intervention arms. When all potential confounders have been identified, these diagnostics help evaluate the degree to which the estimates can be trusted. However, these methods were developed for studies that involved relatively few simultaneous intervention arms and, being iterative in nature [12], they do not scale well to many modern observational datasets that can involve thousands of simultaneous arms.

In recent years several automated causal inference methods have been reported that demonstrate high accuracy in estimating treatment effects on semi-synthetic datasets where the ground truth is known. Some approaches extend logistic regression models [13, 14] to provide covariate balance. Bayesian Additive Regression Trees [15] (BART) have been used for causal modelling [16] and have demonstrated superior performance on benchmark datasets [17]. With recent interest in using deep neural networks for predictive modelling [18], they have been deployed in the causal inference context as well. Shi et al. [19] and Alaa et al. [20] have used them for joint modeling of propensity scores as well as conditional outcomes. Others have focused on using neural networks to estimate effects of counterfactual treatments without estimating propensity scores [21, 22]. Causal inference using neural-network-based generative models has also been demonstrated [23, 24]. While some of the deep learning architectures mentioned above generate propensity scores [19, 20], their use in conjunction with popular techniques like IPTW or DR for computing average treatment effects (ATE) and assessing bias removal has not been demonstrated. Also, the use of these methods in situations where there are a large number of intervention arms has not been explored.

In this study, we developed a method we call Balancing Covariates Automatically Using Supervision (BCAUS) that is directly compatible with trusted protocols to estimate treatment effects and diagnose the quality of those estimates, while making these approaches automatically scalable to an arbitrary number of simultaneous intervention arms without any need for manual iteration. Our approach removes manual iteration through the training of deep neural networks for propensity modelling that explicitly remove covariate imbalance [19, 20]. This is accomplished by training networks with a joint loss function which penalizes both the incorrect prediction of the assigned treatment as well as the degree of imbalance between inverse probability weighted covariates of intervention arms.

We compared BCAUS with multiple state-of-the-art approaches on two clinical datasets. First, on the Infant Health and Development Program (IHDP), a common public semi-synthetic dataset where the ground truth was known, to assess parity in the accuracy of estimation of the ATE. Second, on a private real-world dataset for Type-2 diabetes, which involved 133 simultaneous intervention arms, to assess scalability. While the ground truth ATEs on the diabetes dataset are unknown, we analyzed similarities between the estimations of BCAUS and BART. We found that BCAUS had competitive accuracy for estimating synthetic treatment effects and provided highly concordant estimates on the real-world dataset but was an order-of-magnitude faster. We see our work as being complementary to other automated approaches while still being compatible with conventional causal inference workflows.

## Methods

**Fig. 1:**
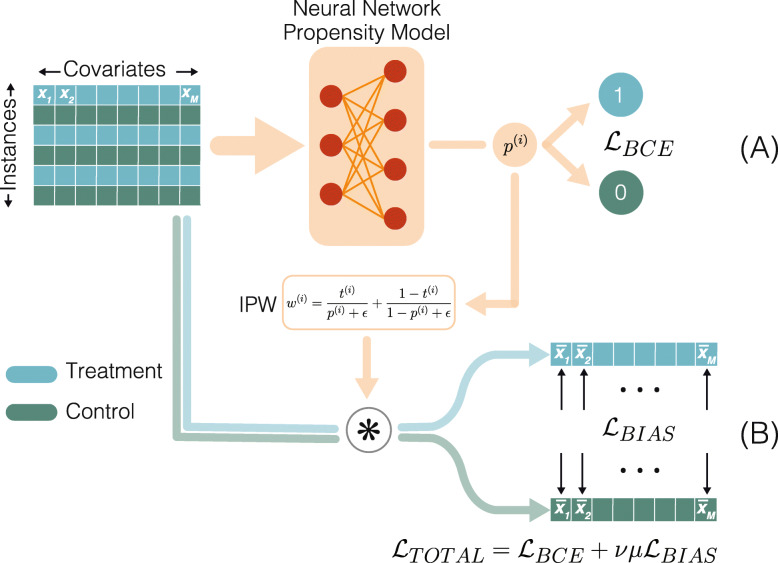
Schematic of BCAUS. Structured data composed of control and treatment instances is fed to a neural network. The output of the network *p*^(*i*)^ for each instance *i* is a propensity score that optimizes a combination of two loss functions. **A** The binary cross entropy loss ${\mathcal{L}}_{BCE}$ is computed by comparing *p*^(*i*)^ against targets *t*^(*i*)^ = 0 for control and *t*^(*i*)^ = 1 for treatment. **B** The bias loss ${\mathcal{L}}_{B\mathrm{I} AS}$ is computed as follows: (i) *p*^(*i*)^ is used to compute an inverse probability weight (IPW, orange box) that multiplies all covariates of instance *i* (ii) weighted means ${\overline{x}}_j$ are computed separately for treatment and control groups for each covariate x_*j*_, and (iii) the mean squared error between weighted means of treatment and control covariates defines the bias loss. The sum of both losses is computed and backpropagated. *μ* is the scalar ratio of ${\mathcal{L}}_{BCE}$ to ${\mathcal{L}}_{BIAS}$ that is detached from the computation graph. The relative contribution of each loss component is tuned using hyperparameter, *ν*

The BCAUS method trains a deep neural network with a joint loss term consisting of two components. The first component penalizes the incorrect prediction of treatment assignment as in traditional propensity-score models. The second component depends explicitly on the imbalance or bias between intervention arms in the data, forcing the network to learn parameters that minimize this bias. A schematic of our method is shown in Fig. 1. The propensity model is a binary classifier which takes the covariates of each individual as input and predicts whether they have been assigned to the control group or the treatment group. In our work we use deep neural networks with dropout regularization and rectified linear unit (ReLu) activations as the propensity model. The output of the network, *p*^(*i*)^ (referred to as a propensity score) is trained against targets 0 (for the control group) or 1 (for the treatment group) using a binary cross entropy loss ${\mathcal{L}}_{BCE}$. All covariates of each instance are weighted by its inverse probability weight (IPW) and the mean squared error between treatment and control groups is computed to obtain the bias loss ${\mathcal{L}}_{BIAS}$. The losses ${\mathcal{L}}_{BCE}$ and ${\mathcal{L}}_{BIAS}$ can have very different scales and their relative magnitudes may vary batch-to-batch during training. To account for this and to ensure that the contributions of the two components can be precisely tuned, for each training batch we compute a scalar ratio of the losses, $\mu ={\mathcal{L}}_{BCE}/{\mathcal{L}}_{BIAS}$ that is detached from the computation graph i.e. gradients are not computed for *μ* during backpropagation. The total loss term for training the network is:

1 $$ {\mathcal{L}}_{TOTAL}={\mathcal{L}}_{BCE}+\nu \mu {\mathcal{L}}_{BIAS} $$

The hyperparameter *ν* can be adjusted to tune the relative contributions of the two modeling objectives. The cross-entropy loss is computed as:

2 $$ {\mathcal{L}}_{BCE}=\sum \limits_i{t}^{(i)}\mathit{\log}\left({p}^{(i)}\right)+\left(1-{t}^{(i)}\right)\ \mathit{\log}\left(1-{p}^{(i)}\right) $$

Here *t*^(*i*)^ ∈ {0, 1} is the treatment given to individual *i*. To compute the bias loss, the propensity score *p*^(*i*)^ is used to compute the IPW:

3 $$ {w}^{(i)}=\frac{t^{(i)}}{p^{(i)}+\epsilon }+\frac{1-{t}^{(i)}}{1-{p}^{(i)}+\epsilon } $$

Here *ϵ* is a small positive number used to stabilize the denominator. The mean squared error of the *M* covariates weighted according to Eq.3 is used to calculate the bias loss:

4 $$ {\mathcal{L}}_{BIAS}=\frac{1}{M}\sum \limits_{j=1}^M{\left(\frac{\sum \limits_i{t}^{(i)}{w}^{(i)}{x}_j^{(i)}}{\sum \limits_i{t}^{(i)}{w}^{(i)}}-\frac{\sum \limits_i\left(1-{t}^{(i)}\right){w}^{(i)}{x}_j^{(i)}}{\sum \limits_i\left(1-{t}^{(i)}\right){w}^{(i)}}\right)}^2 $$

The two terms in the Eq. 4 represent the weighted means of the covariates for the treatment and control groups respectively. The bias loss ${\mathcal{L}}_{BIAS}$ is not calculated against a fixed ground-truth label as is the case for the cross-entropy loss, ${\mathcal{L}}_{BCE}$. However, being composed entirely of differentiable elements, gradients of this loss can be computed efficiently via backpropagation.

To assess balance, the standardized difference Δ_*j*_ for the covariate *x*_*j*_ is computed according to:

5 $$ {\Delta}_j=\frac{\left|{\overline{x}}_{j, treatment}-{\overline{x}}_{j, control}\right|}{\sqrt{\left({s}_{j, treatment}^2+{s}_{j, control}^2\right)/2}} $$

Here ${\overline{x}}_j$ is the weighted mean of *x*_*j*_ and ${s}_j^2$ is its weighted variance with weights assigned according to Eq. 3. The standardized difference can also be defined for the raw data without the weights, in which case ${\overline{x}}_j$ and ${s}_j^2$ represent the unweighted mean and variance respectively.

During training we consider a covariate as being balanced if the standardized difference, Δ for the weighted covariate is less than 0.1. Several authors have recommended that the standardized difference be used as a diagnostic in clinical observational studies [25–27] with the value of 0.1 being widely accepted as a sufficient threshold for ensuring the removal of bias between treated populations [28, 29]. We note here that the exact value of this threshold is incidental to our method and any user-specified value may be used; the loss term ${\mathcal{L}}_{BIAS}$ tries to explicitly reduce Δ to 0 for each covariate regardless of this value. We pass the threshold value as a parameter to our training function and use an “early-stopping” strategy where training terminates when Δ < 0.1 for all covariates. While training may proceed in batches, it is crucial that the number of balanced covariates is always evaluated for the entire dataset.

## Results

We demonstrate the use of BCAUS on two datasets: i) The IHDP dataset with a single pair of control and intervention arms that was introduced by Hill [16] and is used as a benchmark dataset in several recent deep-learning causal inference studies [19–24, 30] and ii) A large observational diabetes dataset studying the effects of anti-hyperglycemic medications on hemoglobin-A1c (HbA1c) values. The ground-truth ATE values for the semi-synthetic IHDP dataset are known. We combined BCAUS with IPTW and DR to estimate ATEs and compared them against the ground-truth. Since the diabetes dataset is drawn from real-word evidence, the ground-truth ATE is unknown. For this reason, we compared ATE values estimated by BCAUS-IPTW against those returned by BART. The choice of BART for comparison was motivated by the fact that it was one of the top performers at the 2016 Atlantic Causal Inference competition [17]. As a diagnostic, we also evaluated the specification of all models by comparing standardized differences Δ between covariates before and after BCAUS training.

### IHDP dataset results

The IHDP dataset is a modified version of data from a randomized experiment [31] that was conducted to determine the effect of an interventional program on the cognitive development of pre-term babies. The data consists of 25 covariates (labeled *x*_1_ through *x*_25_) of which 6 are continuous and the rest are binary, a treatment variable, and simulated factual and counterfactual outcomes. The continuous covariates are z-scored to have zero mean and unit variance. By intentionally censoring data from certain subjects, the dataset has been imbalanced to simulate an observational study. To compare results of BCAUS against earlier studies [21–23], we use 1000 realizations of the IHDP dataset made available by Shalit et al. [22]. This dataset was generated by the authors of Ref. 22 using the formulae specified in Ref. 16 such that each realization has a distinct ATE value. While each realization contains a single intervention arm, in aggregate the dataset is analogous to a multi-arm study with 1000 intervention arms. There are 747 individuals in the dataset of whom 608 belong to the control group and 139 belong to the treatment group. The 747 individuals are divided into a training set with 672 individuals and a holdout set of 75 individuals. We subdivided the larger dataset into training (500 samples) and cross-validation (172 samples) sets as in other studies [21, 22] but did not perform any additional transformations.

**Fig. 2:**
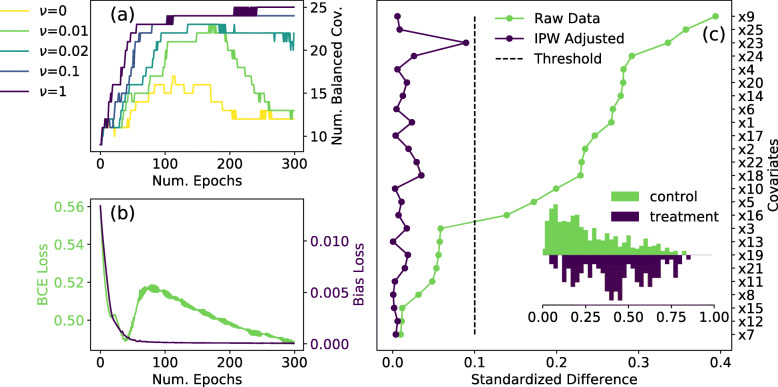
Results of BCAUS on IHDP dataset. Model trained on one realization. **a** Number of balanced covariates (standardized difference, *Δ* between arms < 0.1, Eq. 5) as training progresses for different values of hyperparameter *ν*. When *ν* = 0, the network is trained with ${\mathcal{L}}_{BCE}$ alone. **b** Loss curves for ${\mathcal{L}}_{BCE}$ (green) and ${\mathcal{L}}_{BIAS}$ (violet) for network trained with *ν* = 1. **c** Standardized differences for all 25 covariates in IHDP dataset for network trained with *ν* = 1. Green trace is raw unadjusted data and violet trace shows data adjusted by Inverse Propensity Weights (IPW). Dashed line represents threshold at 0.1. Inset shows normalized histograms of the distribution of propensity scores i.e. output of BCAUS, for control (green) and treatment (violet) groups

The neural network trained on the IHDP data consisted of two hidden layers with 50 neurons each. The output layer was a single neuron which represented the propensity score. We trained a set of 5 models on a single realization of the IHDP dataset with different values of the hyperparameter *ν* to demonstrate the effect the loss term of Eq. 4 has on achieving covariate balance. The results of our experiments are shown in Fig. 2. In Fig. 2(a), we show the number of balanced covariates as training proceeds for different values of *ν*. We observe that when the network is trained with only ${\mathcal{L}}_{BCE}$ i.e. *ν* = 0, only a fraction of the covariates are balanced at the end of training. Higher values of *ν* result in faster convergence. The classifier loss ${\mathcal{L}}_{BCE}$ and the bias loss ${\mathcal{L}}_{BIAS}$ are shown in Fig. 2(b) for the model trained with *ν* = 1. The very different scales of the two losses demonstrates the importance of the parameter *μ* in the loss function. Figure 2 (c) shows the standardized difference Δ between treatment and control, with and without inverse propensity weight (IPW) adjustment, for the model trained with *ν* = 1. For each of the 25 covariates the adjusted difference lies below 0.1 indicating that the propensity model is well specified. The inset of Fig. 2 (c) shows normalized histograms of the distribution of propensity scores, i.e. output of BCAUS, for treatment and control groups. The large degree of overlap between the distributions is essential for matching-based methods that may be used downstream to estimate the ATE.

To compare against previous work on neural-network-based causal inference methods, we evaluated BCAUS on 1000 realizations of the IHDP dataset. For each realization we trained BCAUS on 500 training samples and computed the ATE using IPTW and DR. For DR, we trained two linear regressors (for control and treatment respectively) with an L2 penalty and chose the regularization parameter by performing 3-fold cross-validation on the training set. We computed the mean absolute error between the estimated and ground-truth ATEs, *ϵ*_*ATE*_ across 1000 realizations and picked BCAUS hyperparameters to minimize this value on 172 cross-validation samples. Table 1 reports *ϵ*_*ATE*_ between estimated ATEs and ground-truth ATEs. The in-sample *ϵ*_*ATE*_ is computed by combining the 500 training and 172 cross-validation examples and the out-of-sample value is computed on 75 held out examples. We observe that the performance of BCAUS is comparable to other neural-network-based methods and can complement these approaches. We provide code in the Supplementary Information section to reproduce BCAUS results on this data.

**Table 1:** Comparing BCAUS against other deep-learning-based causal inference algorithms

Method	In-sample ***ϵ***_***ATE***_	Out-of-sample ***ϵ***_***ATE***_
BNN	0.37 ± .03	0.42 ±.03
BLR	0.72 ±.04	0.93 ±.05
TARNet	0.26 ±.01	0.28 ±.01
CFR MMD	0.30 ±.01	0.31 ±.01
CFR WASS	0.25 ± .01	0.27 ±.01
GANITE	0.43 ± .05	0.49 ± .05
Dragonnet	0.14 ± .01	0.21 ± .01
CEVAE	0.34 ±.01	0.46 ±.02
BART	0.47 ±.02	0.66 ±.03
BCAUS IPTW	0.30 ± .01	0.60 ±.02
BCAUS DR	0.13 ±.00	0.29 ±.01

We include BART for comparison even though it is not neural network based. ϵ_ATE_ (lower is better) is the mean absolute error between estimated ATE and ground-truth ATE. *BNN* Balancing Neural Network [21], *BLR* Balancing Linear Regression [21], *TARNet* Treatment-Agnostic Representation Network [22], *CFR* Counterfactual Regression [22], *GANITE* Generative Adversarial Nets for inference of Individualized Treatment Effects [24], Dragonnet [19], *CEVAE* Causal Effect Variational Autoencoder [23], *BART* Bayesian Additive Regression Trees [16]. In-sample value is computed on 672 examples (training + cross-validation) and the out-of-sample value is computed on 75 examples in the hold-out set. The standard error across 1000 realizations is reported as the uncertainty. Performance of BCAUS is comparable to other models

### Diabetes dataset results

The diabetes dataset consists of data drawn from health insurance claims of 140,000 patients with Type-2 diabetes mellitus (T2DM) that was collected over a 5-year time period between 1st January 2015 and 31st December 2019. Individuals are included in the dataset if they have a medical claim indicating T2DM (ICD code E11), a history of high levels of glycated hemoglobin (HbA1c > = 9%), and prescription claims for anti-hyperglycemic medications. The data tracks the effect of anti-diabetic drugs on HbA1c values and can be used to estimate the comparative effectiveness of individual drugs or combinations of drugs in real-world settings. The data consists of the following 21 covariates: age, gender, 15 variables indicating the presence or absence of comorbid conditions defined by the Charlson Comorbidity Index [32], and 4 variables describing the racial makeup and income levels in the patient’s zip code tabulation area (ZCTA). Data on the last four come from US census information [33]. Of these 21 covariates, age, and the four ZCTA variables are continuous while the rest are binary. In addition, a treatment variable denotes which anti-diabetic drug or combination was used to treat the individual. The outcome variable is the change in HbA1c value from baseline in a 6–12 month period following treatment. Drugs are identified by their therapeutic class names alone (e.g. GLP-1 Agonists, SGLT2 Inhibitors, etc.). In many instances, patients shift treatment regimens during the 5-year time frame. As a result, in the filtered dataset 289, 000 treatment assignments are observed. Only those treatments where the size of the cohort is greater than 30 are retained. There are 134 unique drug combinations that meet this criterion.

We trained BCAUS on the diabetes dataset setting Insulin (the most common treatment in the dataset) as control and comparing all other drug combinations against this control. We did not explicitly model for time-varying effect but instead considered each treatment-assignment as belonging to a different subject in the study. We used neural networks with two hidden layers of 42 neurons for the BCAUS model. The maximum number of epochs was set to 1000, but an early-stopping procedure was implemented where training terminated if all 21 covariates remained balanced for more than 10 epochs. The hyperparameter *ν* was set for each control-treatment pair using stratified k-fold cross-validation, where the network was trained on k-1 folds and the number of balanced covariates (the primary metric to be optimized) were counted for the k-th fold. The number of folds was set to 3. Once an optimal value of *ν* was obtained, the number of balanced covariates was counted on the entire dataset combing all folds.

**Fig. 3:**
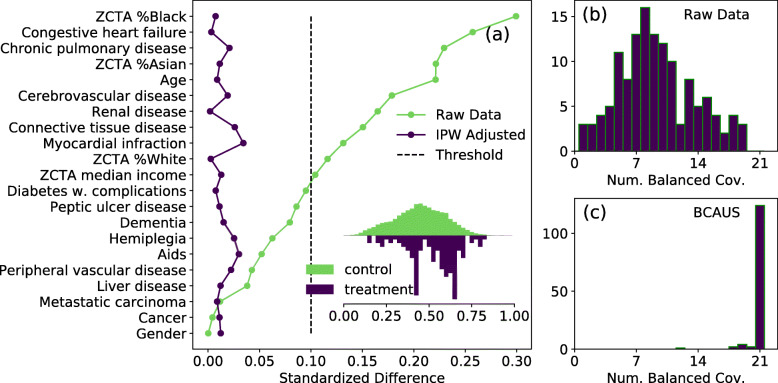
Results of BCAUS on Diabetes dataset. **a** Standardized differences for all 21 covariates in Diabetes dataset for one arm: Treatment = Insulin + Metformin + GLP-1 Agonist + SGLT2 Inhibitor + Sulfonyurea; Control = Insulin. Cohort sizes are 125 and 44,600 respectively, implying a class-imbalance ratio ~ 1:350. Green trace is raw unadjusted data and violet trace shows data adjusted by IPW. Dashed line represents threshold at 0.1. Inset shows normalized histograms of the distribution of propensity scores i.e. output of BCAUS, for control (green) and treatment (violet) groups. ZCTA = Zip-Code Tabulation Area. **b** Histogram of number of balanced covariates (standardized difference, *Δ* < 0.1 between control and treatment) in each intervention arm prior to BCAUS training and IPW adjustment. Shown here for 133 intervention arms. **c** Same as (**b**), but after BCAUS training and adjustment. For a majority of intervention arms (124 of 133) all covariates are balanced

In real-word settings such as this, it is not uncommon to observe extreme differences between treatment and control cohort sizes. In our example, the Insulin-treated control group had a cohort size of 44,600, while the median cohort size of the 133 treatment groups was 163. From a machine learning perspective, such class imbalances in binary classification tasks are usually treated by sub-sampling or by loss weighting. In experiments described below, we do not use either of these techniques. Training with the joint loss of Eq. 1 is observed to adequately correct for class imbalance. As an example of a treatment cohort with large class imbalance, we consider treatment by a combination therapy of Insulin + Metformin + GLP-1 Agonist + SGLT2 Inhibitor + Sulfonyurea. In this case there were only 125 examples in the treatment cohort, representing a treatment-to-control cohort size ratio of approximately 1:350. As shown in Fig. 3(a) BCAUS was able to remove covariate imbalance present in the unadjusted raw data. The inset of Fig. 3(a) shows normalized histograms of the distribution of propensity scores. To measure the performance of BCAUS on all 133 arms, we count the number of covariates which have low bias (Δ < 0.1) for each arm in the raw data prior to BCAUS training and adjustment. A histogram is shown in Fig. 3(b). We see that in none of the 133 arms are all 21 covariates balanced. The median number of balanced covariates is 9. In Fig. 3(c), we show the same histogram but after BCAUS training and adjustment. In a majority of cases (124 of 133) all 21 covariates are balanced. For a small number of intervention arms (8 of 133) BCAUS is able to balance ≥ 18 covariates but not all 21. These intervention arms tended to have very few covariates balanced prior to training and adjustment (left tail of distribution in Fig. 3(b)). For one intervention arm which had only 2 covariates balanced in the raw unadjusted data, BCAUS was able to balance only 12 covariates.

**Fig. 4:**
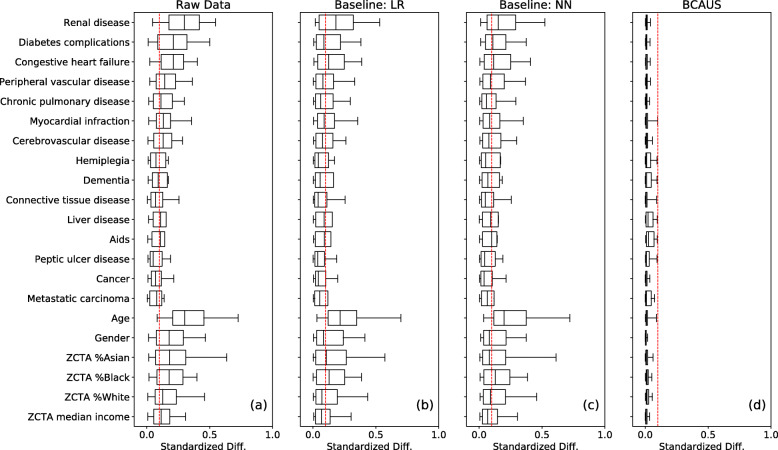
Comparison between BCAUS and baseline logistic regression and neural network propensity score models on the diabetes dataset. For every covariate, box plots show distributions of standardized differences, Δ between control and treatment cohorts for 133 intervention arms. Whiskers are at 5 and 95 percentiles. Red dashed lines show the threshold at 0.1 below which covariates are considered balanced. **a** Raw unadjusted data without IPW weighting. **b** Covariates weighted with IPWs from baseline logistic regression (LR) model. **c** Covariates weighted with IPWs from baseline neural network (NN) model. **d** Covariates weighted with IPWs from BCAUS

We measured covariate imbalance for all 133 intervention arms before and after IPW adjustment comparing BCAUS against two baseline models: (i) logistic regression and (ii) a neural network classifier trained only to predict treatment assignment. We chose logistic regression as a baseline because it is often used for propensity modeling in observational studies, while the neural network was chosen to emphasize that a non-linear model does not automatically guarantee covariate balance. To make the comparison fair, we used the same model architecture (number of layers, neurons etc.) for the baseline neural network model as BCAUS. Note that this can also be achieved simply by setting the hyperparameter *ν* to zero. Both baseline models were used “out-of-the-box” in that no attempt was made to iteratively tweak them if covariate balance was not achieved. Results of this comparison are shown in Fig. 4. Each box plot shows the distribution of standardized differences Δ (between treatment and control arms) for a particular covariate for all 133 intervention arms. The red dashed line shows the 0.1 threshold level. The left panel shows the unadjusted data and we observe that large imbalances exist in the covariates. When weighted by IPWs from the logistic regression model or the neural network model, covariate imbalance is reduced as shown in panels (b) and (c) respectively. However, a much more dramatic reduction in imbalance is seen in the BCAUS model as shown in panel (d). This demonstrates that BCAUS is effective at balancing covariates in real world datasets with an extremely large number of intervention arms and outperforms common baselines.

**Fig. 5:**
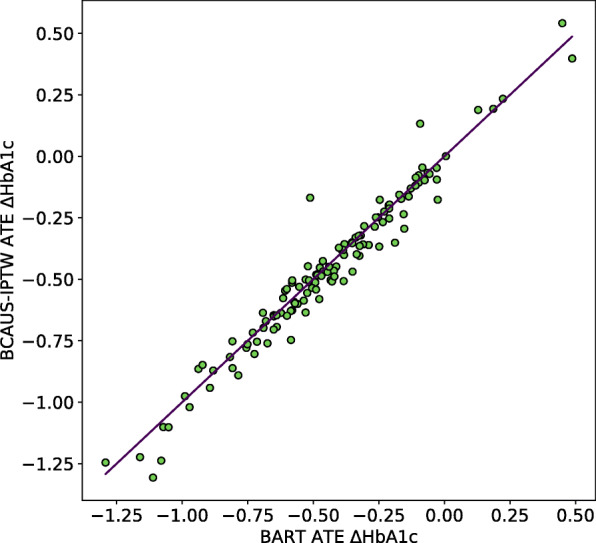
Comparison between BCAUS and BART ATE estimates on the diabetes dataset. Since ground truth for the diabetes dataset is unknown, we compare BCAUS estimates versus BART estimates. ATE estimated using BCAUS-IPTW plotted against ATE estimates from BART (green dots) for 133 intervention arms. Units are percentage changes in glycated hemoglobin i.e. HbA1c. Straight line represents y = x. Mean absolute error between the two estimates is 0.04

We computed ATEs using propensity scores from BCAUS using IPTW for all intervention arms with 21 balanced covariates and compared it against ATE values from BART trained using the default specifications recommended by Chipman et al. [15]. Figure 5 plots BCAUS ATE values against BART ATE values for the 133 intervention arms. We observe good agreement between the two methods with a mean absolute error between the two estimates of 0.04. While the results of the two models are comparable, it is not possible to say which one is more accurate because the ground truth ATEs for this real-world dataset are unknown. Since BART is regression based and not propensity-score-based, we do not compare BCAUS and BART in terms of their ability to achieve covariate balance. We note here that since BART relies on Monte Carlo sampling, we found it to be substantially slower than BCAUS with a run time of approximately 29 h compared to 50 min for BCAUS.

## Discussion

The estimation of causal effects is essential in nearly every field as correct estimation empowers us to optimize decision making based on evidence. Large observational datasets that are becoming increasingly common in healthcare and medicine may have a multitude of intervention arms and require causal inference techniques that scale appropriately. While causal inference workflows that are automated are critical, it is also desirable that these methods be fast and compatible with more conventional workflows so that their results can be trusted. Many recently proposed deep-learning-based methods are highly automated and fast. BCAUS complements these algorithms and at the same time utilizes methods and diagnostics that are commonly used for observational studies in medicine.

On the semi-synthetic IHDP benchmark where ground-truth is known, BCAUS was able to correctly estimate the ATE to within an error that was comparable to other deep-learning-based methods. Of the models considered for comparison in Table 1 BNN, BLR, CFR and TARNet use neural networks to learn representations of the baseline covariates and train regression models on these learned representations. A comparison of the performance of these models with BART and other non-neural-network-based methods has been reported by Shalit et al. [22]. GANITE and CAVAE use generative adversarial networks and variational autoencoders respectively to learn the probability distributions of the covariates and outcomes and to estimate individual treatment effects. These methods do not generate propensity scores and cannot be used in conjunction with IPTW or DR methods. The Dragonnet method, on the other hand, models both treatment assignment as well as outcomes and is very similar to BCAUS DR. While Draggonet uses a neural network for outcome modeling, BCAUS DR, as demonstrated here, used a simple linear regressor for this task. The difference in performance between the two methods may be attributed to the more flexible modeling scheme used in Dragonnet. We also observed a difference in performance between BCAUS IPTW and BCAUS DR that suggests that despite balancing all covariates there was some residual misspecification in the propensity-score model that was corrected in the DR framework by the regression model. Setting a tighter balancing threshold (lower than 0.1) and training the network for longer may help reduce the difference between the two estimates.

On the real-world diabetes dataset involving significant covariate imbalance as well as class imbalance across hundreds of simultaneous intervention arms, BCAUS demonstrated better performance at reducing imbalance than either logistic regression or neural network propensity models. The ground-truth ATEs are unknown for this dataset, but BCAUS estimates using IPTW were comparable to BART estimates. In our runs we found BCAUS to be ~ 30 times faster than BART at analyzing the 133 intervention arms in the diabetes dataset.

While BCAUS was able to generate correctly specified propensity models for a majority of intervention arms in the diabetes dataset, in a small number of cases all covariates could not be balanced. The loss term ${\mathcal{L}}_{BIAS}$ attempts to match the first moments of IPW adjusted covariates. Supplementing this loss with terms which match higher moments [14] may ameliorate this issue. The effectiveness of the bias loss at correcting class imbalance also needs further investigation. This loss term assigns equal importance to imbalance in each covariate. If it is important to reduce imbalance in certain covariates more than in others, the mean squared error may be replaced by a weighted mean of squared errors where weights are assigned by the modeler in proportion to the relative importance of each covariate. While not explored in the present manuscript, BCAUS may also be used for time-varying confounding. In this case the propensity score output by BCAUS can be used to compute inverse propensity weights that may then be used to solve for the appropriate estimating equations of the marginal structural model under consideration. We will report on these extensions of BCAUS in future publications.

## Conclusion

BCAUS can generate correctly specified propensity models for curated benchmark datasets as well as far-from-ideal, real-world datasets. However, to get accurate estimates of the causal effect, it is essential that all potential confounders are identified for each arm of the study using subject-matter expertise and only confounding covariates are used for propensity score modeling. When used in conjunction with well-established causal-inference techniques it can match the performance of recently proposed neural network methods. It scales well to cases where there are numerous intervention arms, where class imbalance is severe, and where traditional techniques of iterating between model tuning and covariate balance testing are impractical. It is our expectation that BCAUS will automate and speed up current causal inference modeling approaches in medicine and enable the design of massive multi-arm studies that were previously infeasible.

## Data Availability

Data availability The diabetes data are not publicly released due to HIPAA regulations and patient privacy. The corresponding author may be contacted to discuss data access following approved agreements. The IHDP benchmark datasets can be generated by using the NPCI package [34], however confusion exists on the setting used in Shalit et al. [22] (see footnote on page 7 of Shi et al. [19]). In the interest of reproducibility, we used data made available by Shalit et al. [22] These datasets are available online at: Train + CV 672 samples: http://www.fredjo.com/files/ihdp_npci_1-1000.train.npz.zip Hold-out 75 samples: http://www.fredjo.com/files/ihdp_npci_1-1000.test.npz.zip Code availability Code for BCAUS training and evaluation on the IHDP benchmark has been made available as Supplementary Material with the manuscript and will be hosted online at https://github.com/gstef80/bcaus_nma. The repository will maintain the code referenced here and also offer additional enhancements and extensions as they develop.

## Abbreviations

BCAUS: Balancing Covariates Automatically Using Supervision
RCT: Randomized Control Trial
FDA: Food and Drugs Administration
IPTW: Inverse Probability of Treatment Weighting
DR: Double Robust
BART: Bayesian Additive Regression Trees
ATE: Average Treatment Effect
IHDP: Infant Health and Development Program
ReLu: Rectified Linear Unit
IPW: Inverse Propensity Weight
BCE: Binary Cross Entropy
T2DM: Type-2 Diabetes Mellitus
HbA1c: Hemoglobin A1c
ICD: International Classification of Diseases
ZCTA: Zip Code Tabulation Area
GLP-1: Glucagon-Like Peptide 1
SGLT2: Sodium-Glucose Cotransporter 2
LR: Logistic Regression
NN: Neural Network
